# The Structure, Composition, and Role of Periplasmic Stator Scaffolds in Polar Bacterial Flagellar Motors

**DOI:** 10.3389/fmicb.2021.639490

**Published:** 2021-03-11

**Authors:** Xiaotian Zhou, Anna Roujeinikova

**Affiliations:** ^1^Infection and Immunity Program, Monash Biomedicine Discovery Institute, Monash University, Clayton, VIC, Australia; ^2^Department of Microbiology, Monash University, Clayton, VIC, Australia; ^3^Department of Biochemistry and Molecular Biology, Monash University, Clayton, VIC, Australia

**Keywords:** bacterial flagellar motor, structure and function, polar flagellum, torque, electron cryotomography

## Abstract

In the bacterial flagellar motor, the cell-wall-anchored stator uses an electrochemical gradient across the cytoplasmic membrane to generate a turning force that is applied to the rotor connected to the flagellar filament. Existing theoretical concepts for the stator function are based on the assumption that it anchors around the rotor perimeter by binding to peptidoglycan (P). The existence of another anchoring region on the motor itself has been speculated upon, but is yet to be supported by binding studies. Due to the recent advances in electron cryotomography, evidence has emerged that polar flagellar motors contain substantial proteinaceous periplasmic structures next to the stator, without which the stator does not assemble and the motor does not function. These structures have a morphology of disks, as is the case with *Vibrio* spp., or a round cage, as is the case with *Helicobacter pylori*. It is now recognized that such additional periplasmic components are a common feature of polar flagellar motors, which sustain higher torque and greater swimming speeds compared to peritrichous bacteria such as *Escherichia coli* and *Salmonella enterica*. This review summarizes the data available on the structure, composition, and role of the periplasmic scaffold in polar bacterial flagellar motors and discusses the new paradigm for how such motors assemble and function.

## Overview of the Bacterial Flagellum

**Figure 1:**
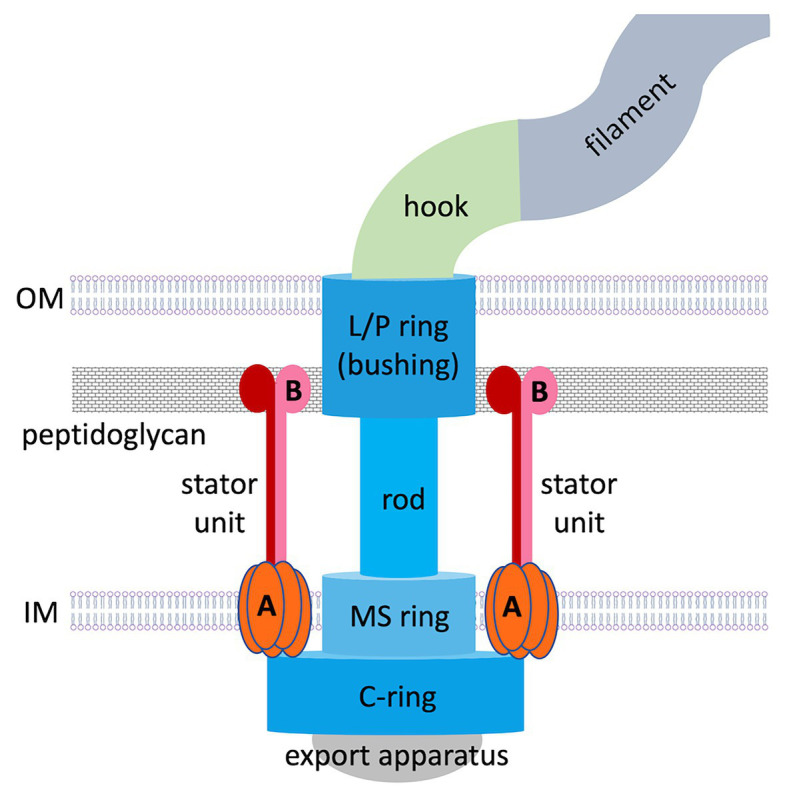
Overall structure of a prototypical flagellar motor in Gram-negative bacteria. Basal body components are colored in shades of blue. Stator components (**A**, MotA; **B**, MotB) are colored in shades of red.

The flagellum (Figure 1) comprises the basal body, hook, and filament. The basal body functions as a rotary motor; the turning force (torque) generated by it is transmitted through the hook to the filament, causing it to spin (Zhao et al., 2014; Carroll and Liu, 2020; Takekawa et al., 2020). Four main types of flagellar arrangement have been observed: monotrichious bacteria (e.g., *Vibrio cholerae*) carry a single polar flagellum; amphitrichous cells (*Campylobacter jejuni*) have one or more flagella at both poles; lophotrichous bacteria (*Helicobacter pylori*) have multiple flagella at one pole; while peritrichous bacteria (*Escherichia coli*) possess multiple flagella distributed over the cell envelope (Schuhmacher et al., 2015).

The flagellar motor is a remarkable nanoscale molecular engine that self-assembles in the cell wall from many protein components. Current knowledge about its structure and function has been largely acquired from studies on peritrichous bacteria. Flagellar assembly begins with the membrane/supramembrane (MS) ring, export apparatus and switch complex [also known as the cytoplasmic ring (C-ring); Kubori et al., 1992; Li and Sourjik, 2011]. Then the rod and hook are assembled by transporting their components *via* the export apparatus (Minamino et al., 2008), a bushing for the rod [comprising peptidoglycan (P) and outer membrane (OM; lipopolysaccharide, L) rings] is added, and stator units assemble in a ring around the rotor perimeter.

The stator ring generates torque using an electrochemical gradient of protons or sodium ions across the inner membrane (IM). In proton-motive-force-driven motors, a single stator unit is an asymmetric assembly of five MotA subunits enclosing two MotB subunits (Deme et al., 2020; Santiveri et al., 2020). In sodium-motive-force-driven motors, the stator is composed of PomAB or MotPS complexes of the same stoichiometry. Existing theoretical concepts for why the stator itself does not spin are based on the assumption that it is anchored to the cell wall by MotB/PomB/MotS binding to P (Roujeinikova, 2008; O’Neill et al., 2011; Reboul et al., 2011; Andrews et al., 2017; Deme et al., 2020; Santiveri et al., 2020). However, visualization of the intact flagellar motor in whole cells (*in situ*), made possible by recent advances in electron cryotomography, revealed that polar flagellar motors contain a substantial proteinaceous periplasmic structure next to the stator (Murphy et al., 2006; Liu et al., 2009; Chen et al., 2011; Beeby et al., 2016; Qin et al., 2017), which has a morphology of disks, rings, or a round cage. It is now recognized that additional periplasmic components are a common feature of polar motors, which sustain higher torque and greater swimming speeds compared to peritrichous bacteria. Evidence has emerged that this periplasmic scaffold may serve as another anchoring region for the stator units. This review summarizes the data available of the structure, composition, and role of the periplasmic scaffold in polar bacterial flagellar motors and discusses the new paradigm for how these motors assemble and function.

## Early Studies: The Discovery of Additional Periplasmic Disks

First evidence that polar flagellar motors possess additional periplasmic components that may be required for their function emerged from electron microscopy (EM) studies of bacterial preparations. Coulton and Murray (1977) observed proteinaceous concentric rings positioned laterally to the basal bodies in *(Aqua)spirillum serpens*. These rings formed 90-nm diameter disks associated with the periplasmic face of the OM. Subsequently, double-layered 90–150-nm diameter disks were observed at the same position in the motors of *C. jejuni* and *Campylobacter* (*Vibrio*) *fetus* subsp. *intestinalis* (Morooka et al., 1983). A similar disk was discovered in the motors of *V. cholerae* (Ferris et al., 1984) and *Wolinella succinogenes* (Engelhardt et al., 1993), but while the diameter of the former did not exceed 41 nm, the average diameter of the latter was 170 nm, suggesting the architecture of this structure is species-specific.

Work on *W. succinogenes* was important, because it showed that the additional disks (from then on referred to as basal disks) are attached not only to the periplasmic face of the OM, but also to the basal body, and that the L/P disk (bushing) is integrated at the center of the basal disk (Kupper et al., 1989; Engelhardt et al., 1993). By that point, studies converged on the hypothesis that the basal disk may serve to anchor the L/P bushing of the motor to the cell wall and ensure correct positioning of the stator units around the rotor.

Interestingly, early work identified basal disks only in proteobacteria with polar flagella, but the possibility that additional periplasmic structures may exist in other flagellated bacteria could not be discounted. The discovery and characterization of such components was hampered by the fact that many are lost in the process of isolation of flagella for analysis by negative staining. It was not until recently that new techniques, such as electron cryotomography and high-throughput genome sequencing, could provide a detailed picture of the entire flagellar motor.

## First Electron Cryotomography Motor Reconstructions: The Discovery and Classification of Periplasmic Scaffolds

The electron cryotomography technique (Oikonomou and Jensen, 2017) has a distinct advantage over traditional transmission EM methods as it allows visualization of the entire flagellar motor in frozen whole cells, without the need for fixation, dehydration, or staining. In 2006, a pioneering study of the spirochaete *Treponema primitia* provided the first 3D reconstruction of the polar motor that included the stator (Murphy et al., 2006). It also revealed a novel periplasmic structure next to the stator, termed the collar, which appears to be unique to Spirochaetes. Furthermore, the observed size of the stator on its periplasmic side could not be accounted for by MotB only, suggesting the presence of some other proteins. It was hypothesized that these extra structures serve as a periplasmic scaffold that recruits, organizes, and stabilizes the stator units. A subsequent electron cryotomography survey of the motor architectures (Chen et al., 2011) and related studies by other labs (Liu et al., 2009, 2010; Raddi et al., 2012) revealed that periplasmic scaffolds exist in the polar motors of many other species, but are absent in the motors of peritrichous bacteria, and that many polar flagellar motors are significantly more complex than the prototypical motors of *E. coli* and *Salmonella enterica*.

**Figure 2:**
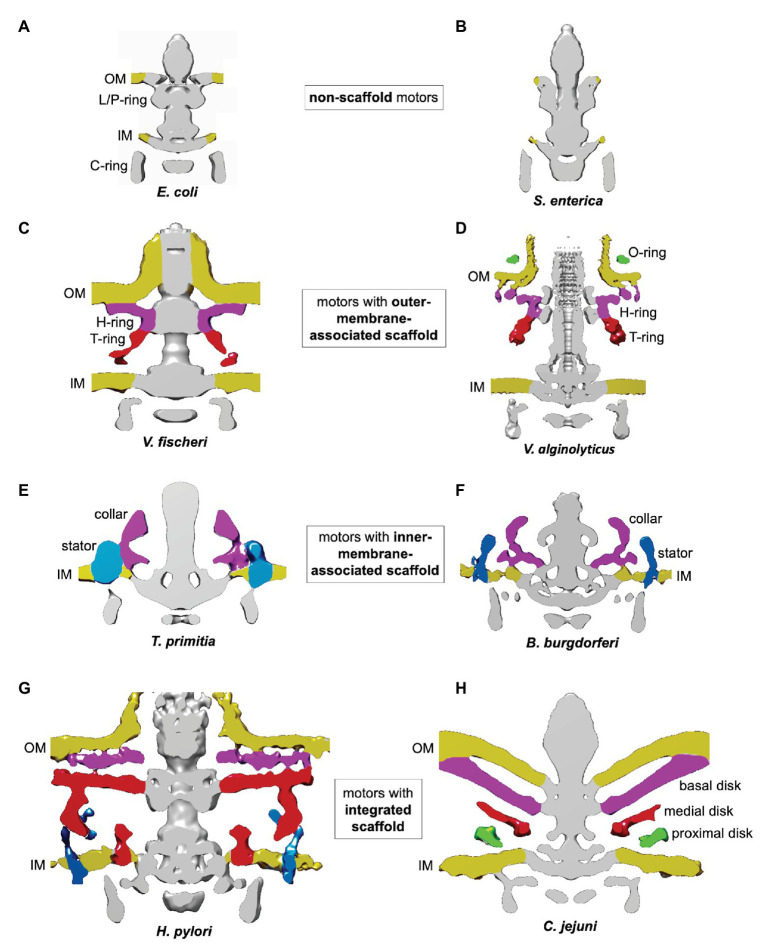
Classification of flagellar motors based on their scaffolds. The figures were prepared using electron cryotomography maps for the flagellar motors from: **(A)** *Escherichia coli* (EMDB-5311; Chen et al., 2011); **(B)** *Salmonella enterica* (EMDB-5310; Chen et al., 2011); **(C)** *Vibrio fischeri* (EMDB 3155; Beeby et al., 2016); **(D)** *Vibrio alginolyticus* (EMDB-21027 and EMDB-21819; Carroll et al., 2020; Zhu et al., 2020); **(E)** *Treponema primitia* (EMDB-1235; Murphy et al., 2006); **(F)** *Borrelia burgdorferi* (EMDB-9122; Qin et al., 2018); **(G)** *Helicobacter pylori* (EMDB-8459; Qin et al., 2017); and **(H)** *Campylobacter jejuni* (EMDB-3150; Beeby et al., 2016).

Flagellar motors can be classified according to four scaffold types: non-scaffold motors (Figures 2A,B); OM-associated scaffold motors (Figures 2C,D); IM-associated scaffold motors (Figures 2E,F); and integrated scaffold motors (Figures 2G,H).

An example of an OM-associated scaffold is the H/T/O-ring system found in the polar motor in *Vibrio alginolyticus* (Zhu et al., 2017, 2020; Figure 2D). The O-ring, associated with the external face of the OM, surrounds the base of the hook. The periplasmic H-ring, associated with the inner face of the OM, surrounds the L/P-ring. The periplasmic T-ring assembles at the outer rim of the H-ring, below the P-ring.

Inner-membrane-associated scaffolds have been found in polar motors driving the periplasmic flagella in Spirochaetes (Murphy et al., 2006; Liu et al., 2009, 2010; Raddi et al., 2012). They are exemplified by the collar-like structures seen in *T. primitia* (Figure 2E) and *Borrelia burgdorferi* (Qin et al., 2018; Figure 2F). The narrow part of these structures is embedded into the IM between the MS and stator rings, while the wider rim is positioned in the periplasm.

An integrated scaffold, spanning the periplasm and associated with both OM and IM, has been found in the polar motors of the closely related bacteria *H. pylori* and *C. jejuni* (Beeby et al., 2016; Qin et al., 2017; Figures 2G,H). The core of the scaffold is a round cage-like structure encircling the rod, the MS, and L/P rings. It is anchored to the IM on one side, and extends to the basal disk associated with the periplasmic face of the OM on the other.

## Molecular Composition of Periplasmic Scaffolds

The OM-associated scaffold of the *Vibrio* spp. motor has been dissected in detail. The T-ring comprises MotX and MotY (Terashima et al., 2006), while the adjacent H-ring is made up of the OM lipoproteins (OMLPs) FlgO and FlgP and the periplasmic protein FlgT (Terashima et al., 2013; Beeby et al., 2016; Zhu et al., 2018, 2020). The protein make-up of the O-ring is not yet known.

The IM-associated scaffold of the spirochaetal motor has been studied extensively in *B. burgdorferi*. The base of the collar contains FlbB (Chen et al., 2011; Moon et al., 2016), which is anchored to the IM *via* its N-terminal transmembrane helix. Although the full protein composition of the collar is not yet known, the tetratricopeptide repeat (TPR) proteins BB0236 (Moon et al., 2018) and FlcA (Xu et al., 2020) have been identified as putative collar proteins that assemble onto the FlbB base.

The molecular composition of the integrated scaffold of the *H. pylori* motor is yet to be established. However, it is in a similar position to the integrated scaffold seen in *C. jejuni* (Figures 2G,H), and it is likely that the two scaffolds share at least some common components. The three main parts of the *C. jejuni* scaffold are: the basal disk associated with the periplasmic face of the OM; a medial disk around the rod; and a disk proximal to the IM (Figure 2H). The basal disk is formed by the OMLP FlgP (homologous to *Vibrio* FlgP), likely in complex with FlgQ (Beeby et al., 2016). The medial disk is composed of paralyzed flagellum protein A (PflA), a periplasmic TPR protein. The IM-proximal disk contains the TPR protein PflB, which is anchored to the IM *via* a single transmembrane helix. Proteins homologous to *C. jejuni* FlgP, PflA, and PflB are present in *H. pylori* (Rajagopala et al., 2007; Sommerlad and Hendrixson, 2007), suggesting *C. jejuni* and *H. pylori* scaffolds assemble in a similar manner.

## The Role of the Individual Scaffold Components

### OM-Associated Scaffold of the *Vibrio* Motor

*ΔflgT* mutant cells lacked the H-ring and mostly produced periplasmic, rather than native, external flagella (Terashima et al., 2010; Zhu et al., 2018). Most of the H-ring was missing in Δ*flgP* mutant cells, which also lacked external hook/filament structures (Beeby et al., 2016). Thus, one apparent function of the H-ring is to mediate the OM penetration during the flagellum biogenesis. This notion is strengthened by reports that mutations in *flgO* also resulted in a reduced number of cells with external flagella (Martinez et al., 2009; Zhu et al., 2018). The other putative function of the H-ring is to anchor the flagellum to the cell wall, by associating with the OM *via* its medial part containing the OMLP FlgP (Morris et al., 2008), and its outer part, containing the OMLP FlgO (Martinez et al., 2009; Zhu et al., 2018). In addition, the H-ring is required for stator ring assembly (Beeby et al., 2016).

The inner part of the T-ring is formed by MotY (Terashima et al., 2006; Zhu et al., 2017), which is likely anchored to peptidoglycan, as it contains a peptidoglycan-binding motif (Okunishi et al., 1996; Kojima et al., 2008). The stator-proximal edge of the T-ring is formed by MotX (Terashima et al., 2006; Zhu et al., 2017). In the absence of MotX or MotY, bacteria were non-motile (Gosink and Häse, 2000), and the stator units were not recruited to the cell pole (Terashima et al., 2006), indicating that the T-ring is also required for stator assembly.

### Inner-Membrane-Associated Scaffold of the Spirochaetal Motor

*ΔflbB*, Δ*bb0236*, and Δ*flcA* mutant cells had less flagella per cell than the wild type, suggesting that all three putative collar components play an important role in the flagella biogenesis (Moon et al., 2016, 2018; Xu et al., 2020). Furthermore, all three mutants were non-motile, lacked the stator, and produced flagella that were abnormally oriented toward the cell pole rather than to the cell cylinder. This shows that the collar is required for correct orientation of the periplasmic flagella and assembly of the stator in *B. burgdorferi*.

### Integrated Scaffold of the Campylobacterotal Motor

The basal disk in the integrated motor scaffold in *C. jejuni* is thought to play a similar role to that of the H-disk in the OM-associated scaffold in the *Vibrio* motor. Both structures contain the OMLP FlgP, although the *C. jejuni* and *Vibrio* proteins share only limited sequence similarity (Beeby et al., 2016). Deletion of *flgP* resulted in the loss of the disk and loss of functionality in both types of the motor, but in contrast to the *Vibrio fischeri* Δ*flgP* mutant, which lacked external hook/filament structures, the Δ*flgP* mutant of *C. jejuni* assembled normal-looking flagella (Sommerlad and Hendrixson, 2007). Another difference is that in the absence of the H-ring, the remainder of the scaffold (the T-ring) still assembled at least in some *Vibrio* species (Zhu et al., 2018), whereas the loss of the basal ring in *C. jejuni* resulted in the loss of the entire scaffold (Beeby et al., 2016). Despite the differences, the loss of function in Δ*flgP* mutants with either type of motor is attributable to the fact that the stator does not assemble in the absence of the basal ring or H-ring (Martinez et al., 2010).

The medial disk containing PflA is required for assembly of the proximal disk containing PflB, and Δ*pflA* mutants are non-motile (Yao et al., 1994) because their motors lack the stators (Beeby et al., 2016). MotB was shown to incorporate into the motor only in the presence of the proximal ring, supporting the hypothesis that upon assembly into the polar motor of *C. jejuni*, the stator units are anchored to the integrated scaffold *via* PflB.

## Implications For the Mechanism of Generation of Higher Torque in Polar Flagellar Motors

Thus, due to recent advances in electron cryotomography, extensive evidence has emerged that in contrast to peritrichous flagella, polar flagellar motors evolved diverse periplasmic scaffolds around the rotor, without which the stator does not assemble and the motor does not function. It is now widely accepted that upon assembly into the polar motors the stator units are anchored not only to peptidoglycan, but also to these scaffolds, and despite unique differences between the architectures of the OM-associated, IM-associated and integrated scaffolds, commonalities start to emerge with regards to their role in the stator function.

It is now recognized that the number of stator units recruited into the motor determines the total torque: the higher the number, the higher the force (Lele et al., 2013). Furthermore, evidence has emerged that, owing to the presence of the stabilizing scaffolds, stator rings in many polar motors are wider than in peritrichous motors (Chen et al., 2011; Beeby et al., 2016; Qin et al., 2017; Chaban et al., 2018; Chang et al., 2019). We now understand that larger-diameter stator rings not only accommodate more stator complexes, but also place them further away from the axis, resulting in a higher momentum of force produced by each complex, and hence higher overall torque. In peritrichous flagella of *S. enterica* and *E. coli*, at least 11 stator complexes can anchor to the peptidoglycan layer and P-ring (Reid et al., 2006; Hizukuri et al., 2010; O’Neill et al., 2011) and apply force on the 40-nm-diameter C-ring, producing ~1,300 pN nm torque (Reid et al., 2006; Thomas et al., 2006). In comparison, the C-ring in the polar motor of *H. pylori*, for example, is significantly larger (57 nM) and surrounded by half as many stator complexes (18; Qin et al., 2017), which is consistent with the observed higher torque (~3,600 pN nm; Celli et al., 2009). The C-ring in the spirochaetal polar motor is similarly large and surrounded by 16 stator complexes (Zhao et al., 2014; Chang et al., 2019), producing a torque of ~4,000 pN nm (Nakamura et al., 2014). Thus, the accumulated structural and functional data on the periplasmic scaffolds in the polar motors are consistent with their role as platforms that recruit a wider power ring to sustain higher torque.

## Implications For Evolutionary Adaptation

The remarkable structural diversity of scaffolds in polar motors suggests they have evolved from a less complex ancestral motor, composed of the common core components seen today in peritrichous motors, by acquiring accessory proteins (Beeby et al., 2020). The existence of class‐ and genera-specific scaffold components (such as MotX/MotY in *Vibrio*, FlbB in Spirochaetes and PflA/PflB in Campylobacterota) is indicative of distinct evolutionary pathways resulting in motors with mechanical outputs that, when combined with other factors, suit specific habitats. At one end of the torque spectrum is the polar motor of *Caulobacter crescentus*. To survive in its low-nutrient freshwater habitat, *C. crescentus* has evolved an efficient motor with no scaffolding structures (Rossmann et al., 2020) that uses a small stator ring comprising only 11 units. The economically low torque (∼350 pN nm; Li and Tang, 2006) is sufficient to propel the cell due to additional thrust created by the helical motion of the cell (Liu et al., 2014). At the other end of the spectrum is the high-torque motor of *H. pylori*. This microorganism resides within the very viscous mucous layer of the stomach (Hooi et al., 2017) and, apparently through natural selection, demonstrates unusually high motility in viscous media (Hazell et al., 1986). Together with the helical cell shape, the high torque, afforded by the wider stator ring supported by a periplasmic scaffold, allows *H. pylori* locomotion in high-viscosity environment.

The mechanism by which the scaffolds recruit and stabilize stator complexes remains enigmatic. TPR domains often serve as protein-protein interaction scaffolds (Blatch and Lässle, 1999), and their presence in FlbB, PflA, and PflB is consistent with their proposed stator scaffolding role. Dissecting the 3D architecture of the periplasmic scaffolds and unraveling the structural basis for their ability to recruit stator units will be a fascinating and worthy task. Achieving this goal will advance our knowledge about the mechanism of the bacterial flagellar motor, and our understanding of the convergent evolutionary pathways to higher-torque polar flagellar motors.

## Author Contributions

All authors listed have made a substantial, direct and intellectual contribution to the work, and approved it for publication.

### Conflict of Interest

The authors declare that the research was conducted in the absence of any commercial or financial relationships that could be construed as a potential conflict of interest.
